# Simple and direct electrochemical detection of rosmarinic acid in food samples based on nanochannel modified carbon electrode†

**DOI:** 10.1039/d4ra03063j

**Published:** 2024-06-11

**Authors:** Wenbo Chen, Hongjuan Ru, Fei Yan, Xianwei Mo

**Affiliations:** a Guangxi Medical University Cancer Hospital, Guangxi Medical University 71 Hedi Road Nanning 530021 P. R. China moxianwei888@163.com; b School of Chemistry and Chemical Engineering, Zhejiang Sci-Tech University Xiasha Higher Education Zone, 928 Second Avenue Hangzhou 310018 P. R. China yanfei@zstu.edu.cn

## Abstract

The detection of rosmarinic acid (Ros A) in food samples holds major significance. Simple and convenient electrochemical detection of Ros A with high performance remains a challenge. In this work, a nanochannel array-modified carbon electrode was constructed using a simple and convenient approach to achieve highly sensitive electrochemical detection of Ros A in food samples. Through simple electrochemical pre-activation of a glassy carbon electrode (GCE), oxygen-containing functional groups were introduced on the electrode surface (p-GCE). Vertically-ordered mesoporous silica film (VMSF) was stably grown on p-GCE through electrochemical-assisted self-assembly (EASA) without the introduction of another adhesive layer (VMSF/p-GCE). Transmission electron microscopy (TEM) characterization demonstrated the highly ordered structure of VMSF with a nanochannel diameter around 2.7 nm. Both p-GCE and the nanochannels significantly enhanced the electrochemical signals of Ros A on the electrode, exhibiting dual signal amplification. VMSF/p-GCE demonstrated sensitive detection of Ros A with a linear range of 500 nM to 1 μM and 1 μM to 35 μM. The detection limit (DL) was 26 nM. Combining the good anti-fouling and anti-interference properties of the nanochannels, VMSF/p-GCE can achieve direct electrochemical detection of Ros A in food samples. The sensor can be easily regenerated for repeated use. The simple fabrication, high detection sensitivity and selectivity of the sensor make it a new strategy for rapid preparation of high-performance electrochemical sensors.

## Abstract 2

A simple electrochemical sensor was developed by integrating a vertically-ordered mesoporous silica film (VMSF) with a pre-activated glassy carbon electrode (p-GCE) to provide dual signal amplification for sensitive detection of rosmarinic acid.

## Graphical abstract

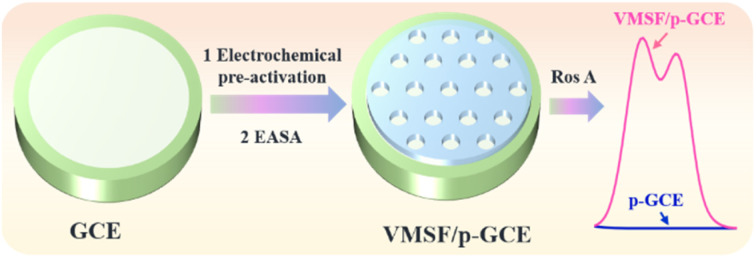

## 1 Introduction

Rosmarinic acid (Ros A) is a natural compound found in the leaves, stems, and flowers of plants belonging to the genus *Rosmarinus*, exhibiting antifungal and antibacterial properties. Due to its inherent antioxidant nature, Ros A finds widespread application in the food industry as an antioxidant to extend the shelf life of food products. Furthermore, Ros A is believed to offer pharmacological benefits such as anti-inflammatory and antioxidant effects. Its pleasant aroma makes it a common ingredient in perfumes, soaps, and skincare products.^1^ In herbal medicine, Ros A is also utilized in traditional medicinal formulations. In recent years, research on Ros A has increased in the fields of medicine and food science, exploring its potential impacts on the nervous system, cardiovascular system, cancer, and other aspects. For instance, Ros A is expected to be effective in preventing Alzheimer's disease due to its inhibition of amyloid-β aggregation.^2,3^ Overall, Ros A, as a multifunctional natural compound, not only plays a role in the food industry but also has garnered widespread attention in the fields of medicine and cosmetics.^4^ However, excessive intake may have adverse effects on human health. Thus, sensitive detection of Ros A holds significant importance.

Currently, methods for detecting Ros A includes physical, chemical, and biological methods. For example, high-performance liquid chromatography (HPLC) is a high-resolution analytical technique widely employed for the detection of Ros A in food and plant extracts.^5–7^ This method allows for the accurate determination of Ros A content and is often coupled with ultraviolet spectrophotometers or mass spectrometers (MS) to enhance analytical precision.^8^ Gas Chromatography (GC) coupled with MS is predominantly utilized for the analysis of volatile compounds of Ros A, particularly suitable for the detection of fragrances and essential oils.^9^ However, both of these chromatographic methods typically require expensive equipment and specialized operational skills. Biological methods, such as enzyme-linked immunosorbent assay (ELISA), achieve the detection of Ros A by binding it with specific antibodies, providing high specificity but potentially requiring a longer analysis time.^10^ In recent years, electrochemical methods have gained significant attention in Ros A detection.^3,11,12^ As natural phenolic acid containing several phenolic hydroxyl groups, Ros A has redox characteristics.^2,3^ By improving electrode materials or introducing molecular recognition elements, the selectivity and sensitivity of electrochemical sensors for Ros A can be enhanced.^13^ However, existing electrochemical sensors often require the introduction of complex and expensive nanomaterials during the electrode modification process, making the operation cumbersome. Additionally, electrochemical detection of complex samples is susceptible to non-specific adsorption of large molecules such as proteins and DNA, as well as interference from co-existing electroactive substances. Challenges still exist in achieving high-performance electrochemical detection of Ros A.

Electrode as well as its potential resolution capability is crucial to the detection selectivity. Carbon-based electrodes, especially glassy carbon electrodes (GCE), have become commonly used sensing electrodes due to their superior conductivity, chemical stability, high mechanical strength, surface modifiability, and biocompatibility.^14–18^ The use of electrochemical pre-activation methods for carbon-based electrode preparation, such as electrochemical polarization, can yield electrochemically and electrocatalytically active carbon electrodes.^19–21^ Typically, electrochemical polarization involves electrochemical polarization of carbon-based electrodes in a conventional electrolyte solution, eliminating the need for complex chemical reagents and cumbersome procedures. In this process, the electrode is initially subjected to a high positive potential for anodic oxidation, followed by cathodic reduction through negative potential or potential scanning. This simple method eliminates the need for complex chemical reagents and tedious operations. The surface of the activated carbon electrode contains abundant edge carbon, defects, and oxygen-containing functional groups, serving as electrocatalytic active sites to enhance potential resolution capability and improve sensor selectivity.^22–24^

Introducing special modification layers, such as molecularly imprinted polymers, nanomaterials, redox conductive polymers or biomolecules, can effectively reduce interference from matrix effects and improve the performance of the electrochemical sensors.^25–29^ Among these, the introduction of vertically-ordered mesoporous silica film (VMSF) as an electrode anti-fouling coating has gained significant attention due to its simple preparation method and unique properties.^30–32^ VMSF features an ordered and vertically aligned nanochannel structure with ultrasmall and uniform pore sizes (typically 2–3 nm), high pore density (up to 7.7 × 10^12^ cm^−2^), adjustable nanoscale thickness (usually 50–200 nm), and excellent chemical and mechanical stability.^33–35^ These characteristics grant VMSF rapid mass transfer capabilities and selective permeability based on size, charge, and hydrophobicity.^36–40^ Therefore, SNF exhibits great potential for the construction of various electrochemical sensors with high sensitivity and good anti-fouling ability.^41,42^

In this work, a method for the rapid preparation of a high-performance electrochemical sensor by integrating VMSF with carbon electrode was developed, which enables sensitive detection of Ros A. The commonly used carbon electrode, glassy carbon electrode (GCE), was pre-treated using electrochemical polarization to create an electrode with high electrochemical activity (p-GCE). Without using any adhesives, VMSF integration was successfully achieved on p-GCE surface (VMSF/p-GCE) through the electrochemical assisted self-assembly (EASA) method to endow the electrode with anti-fouling and anti-interference properties of nanochannel, maintaining excellent adhesion and chemical stability. When VMSF/p-GCE was employed as the sensing electrode, both p-GCE and VMSF enhance the electrochemical signal of Ros A, enabling dual signal amplification to improve the detection sensitivity. Direct electrochemical detection of Ros A in food samples was realized. The fabricated sensor has advantages including simple construction, high sensitivity, good selectivity, and easy regeneration, providing potential for the rapid, convenient and sensitive detection of Ros A.

## 2 Materials and methods

### 2.1 Chemicals and materials

The following materials, including tetraethyl orthosilicate (TEOS), cetyltrimethylammonium bromide (CTAB), potassium hexacyanoferrate(iii) (K_3_[Fe(CN)_6_]), potassium hexacyanoferrate(ii) (K_4_[Fe(CN)_6_]), sodium dihydrogen phosphate dihydrate (NaH_2_PO_4_·2H_2_O), sodium hydrogen phosphate dodecahydrate (Na_2_HPO_4_·12H_2_O), potassium hydrogen phthalate (KHP), rosmarinic acid (Ros A), glucose (Glu), bovine serum albumin (BSA), ascorbic acid (AA), and tartaric acid (Ta), fish sperm molecular DNA (DNA) were purchased from Aladdin Biochemical Technology Co., Ltd (Shanghai, China). Anhydrous ethanol (99.8%) and concentrated hydrochloric acid (HCl, 38%) were obtained from Shuanglin Chemical Reagent Co., Ltd (Hangzhou, China). Sodium chloride (NaCl), calcium chloride (CaCl_2_), potassium chloride (KCl), and zinc chloride (ZnCl_2_) were purchased from Gaojing Fine Chemical Co., Ltd (Hangzhou, China). Rosemary powder and rosemary tea were both acquired from local supermarkets (Hangzhou, China). Phosphate-buffered saline (PBS) was prepared using sodium hydrogen phosphate (Na_2_HPO_4_) and sodium dihydrogen phosphate (NaH_2_PO_4_). All chemicals and reagents were of analytical grade and used without additional treatment. The aqueous solutions employed in the experiments were prepared using ultrapure water (18.2 MΩ cm).

### 2.2 Characterization and instrumentations

The X-ray photoelectron spectroscopy (XPS) test was conducted on the PHI 5300 instrument (PerkinElmer, USA) using Mg Kα source excitation. The morphology and thickness of the VMSF were characterized using a transmission electron microscope (TEM, Hitachi HT7700, Japan). Prior to preparing TEM samples, the VMSF layer was gently scraped from the electrode using a blade, dispersed in anhydrous ethanol under ultrasonication for 40 minutes. The resulting dispersion was then dropped onto the supporting copper grid and air-dried naturally before TEM testing. Electrochemical experiments, including cyclic voltammetry (CV) and differential pulse voltammetry (DPV), were performed on an Autolab electrochemical workstation (PGSTAT302N, Switzerland). All electrochemical experiments utilized a conventional three-electrode system, with Ag/AgCl as the reference electrode, platinum wire or foil as the counter electrode, and either a bare or modified glassy carbon electrode (GCE) as the working electrode. Parameters for DPV measurement included a pulse amplitude of 25 mV, step potential of 5 mV, pulse duration of 0.05 s, and time interval of 0.2 s.

### 2.3 Preparation of p-GCE

GCE (*d* = 3 mm) was sequentially polished with 0.5 μm, 0.3 μm, and 0.05 μm alumina powder, followed by ultrasonic cleaning with ethanol and ultrapure water. The cleaned GCE underwent electrochemical polarization treatment.^43^ Specifically, an anodic oxidation was carried out by applying a constant potential of +1.80 V for 300 s on the GCE. Subsequently, a cathodic reduction was performed in PBS (0.1 M, pH = 7) through cyclic voltammetry scans (scan potential range from −1.30 V to 1.25 V). The resulting pre-activated electrode was denoted as p-GCE.

### 2.4 Fabrication of VMSF/p-GCE

Rapid growth of VMSF on p-GCE was achieved using an electrochemical-assisted self-assembly method (EASA).^44^ A precursor solution for VMSF growth was firstly prepared. A mixture of 20 mL ethanol and 20 mL sodium nitrate (NaNO_3_) solution was combined with cetyltrimethylammonium bromide (CTAB, 1.585 g) and tetraethylorthosilicate (TEOS, 3050 μL), and stirred for 2.5 h at room temperature. Then, the three-electrode system was immersed in the precursor solution, with p-GCE as the working electrode. A constant current density of −0.74 mA cm^−2^ was applied for 10 s. The resulting electrode was quickly removed, thoroughly washed with ultrapure water, and then aged overnight at 80 °C. Then, the electrode with CTAB micelles (SM) in nanochannels was obtained and named SM@VMSF/p-GCE.

### 2.5 Electrochemical detection of rosmarinic acid

Electrochemical detection of Ros A was conducted using PBS (0.1 M, pH = 7.0) as the electrolyte solution. For the analysis of real samples, the content of Ros A in rosemary tea and the extract from rosemary powder were determined. To prepare rosemary tea, 0.5 g of rosemary leaves were added to 15 mL ultrapure water and 35 mL ethanol. The rosemary tea was obtained after 2 hours of ultrasonication treatment. For the preparation of rosemary powder extract, 0.5 g of rosemary powder was added to 15 mL ultrapure water and 35 mL ethanol as the extraction solution. After 2 hours of ultrasonication, the extract from rosemary powder was obtained.

## 3 Results and discussion

### 3.1 Sensor construction through nanochannel modification on pre-activated GCE

**Fig. 1:**
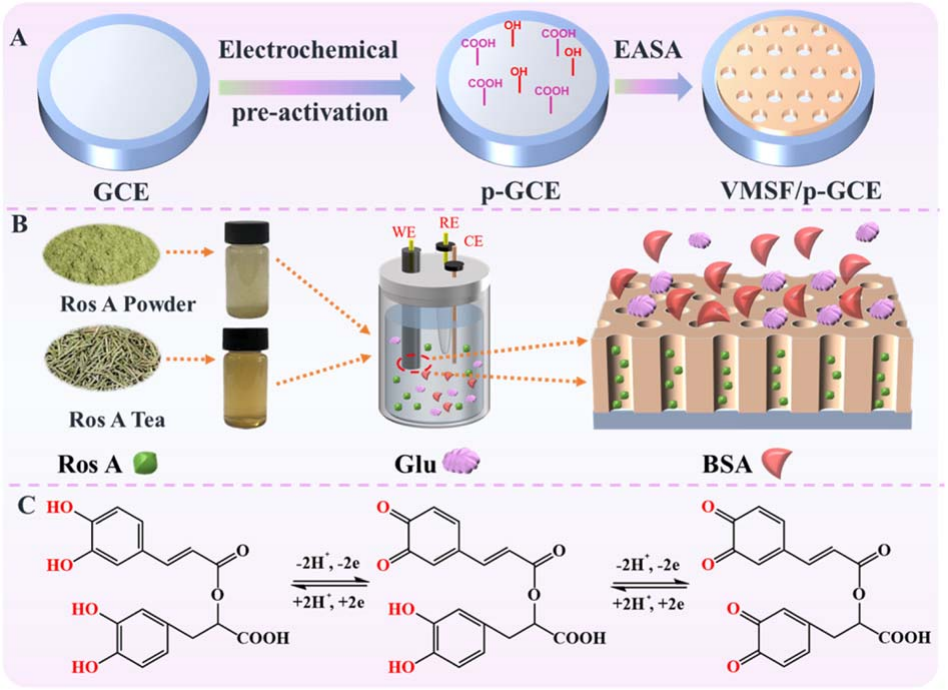
Schematic illustration for the fabrication of VMSF/p-GCE sensor (A), the anti-fouling/anti-interference properties of nanochannel in real sample (B) and the electrochemical process Ros A on electrode (C).

Carbon-based electrodes, known for their excellent conductivity, high chemical stability, adjustable structure, ease of modification, good biocompatibility, and the ability to be repaired or regenerated through simple methods, have found widespread applications in electrochemical sensing. Amongst, glassy carbon electrode (GCE) has been widely adopted in various electrochemical research and applications, becoming the preferred carbon-based electrode material in many laboratories and industrial fields. As illustrated in Fig. 1, GCE was chosen as the carbon-based supporting electrode in this study. However, in the modification of GCE with VMSF, it is necessary to introduce either a binding layer or perform electrode pretreatment, as VMSF cannot directly stabilize on the carbon-based electrode. Nevertheless, introducing a non-conductive binding layer may reduce the electrode's active surface area. There is potential in enhancing VMSF adhesion by surface pretreatment to increase electrode surface activity. As shown, a pre-activated GCE (p-GCE) was obtained through a simple electrochemical polarization process, eliminating the need for introducing a binding layer for stable VMSF modification. This is attributed to the electrochemical polarization introducing abundant oxygen-containing functional groups, such as hydroxyl groups, on the GCE surface.^45^ VMSF is then grown on p-GCE using the electrochemical assisted self-assembly (EASA) method, which involves the growth of silica layers under electrodeposition. Electrodeposition allows for precise control of the deposition rate and thickness by adjusting the current and voltage, resulting in a uniform deposition layer with a fast growth rate.^46–49^ EASA involves applying a negative voltage or current to the electrode, generating hydroxide ions through electrolysis of water, inducing an *in situ* pH gradient on the electrode surface, and thereby facilitating the assembly of surfactant micelles and the sol–gel process of the siloxane precursor. This method enables the simple and rapid growth of VMSF. Simultaneously, the –OH groups on p-GCE can form covalent bonds (–O–Si–O–) with Si–OH groups through a co-condensation reaction, enhancing the adhesion of VMSF. Thus, through a simple electrochemical polarization process, stable modification of VMSF on the GCE surface can be achieved without the need for a special binding layer. After the growth of VMSF is complete, nanochannels modified electrode containing the template surfactant micelle (SM) is obtained (SM@VMSF/p-GCE). After SM is conveniently removed in hydrochloric acid–ethanol solution, VMSF/p-GCE exhibits an open array of nanochannels.

### 3.2 Characterization of p-GCE

**Fig. 2:**
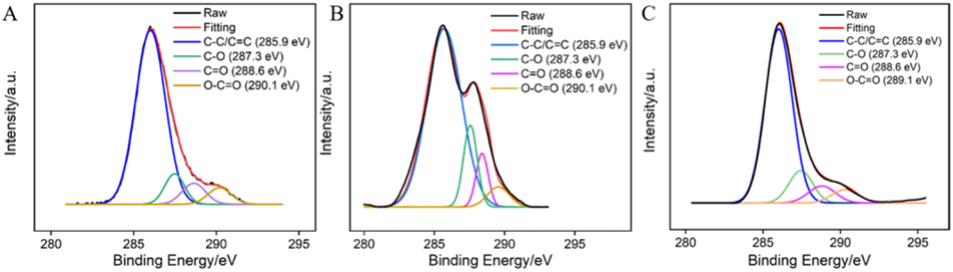
High-resolution C 1s XPS spectra of bare GCE (A), the electrode obtained after anodic polarization (B), and p-GCE (C).

The surface functional groups of GCE before and after electrochemical polarization were characterized through X-ray photoelectron spectroscopy (XPS). The high-resolution C 1s spectra of GCE (A), GCE after anodic polarization (B), and p-GCE (C) are shown in Fig. 2. It can be observed that all three electrodes exhibit four types of carbon bonds, including C–C/C=C (sp^2^ C), C–O, C=O, and O–C=O. After anodic oxidation of GCE, the content of C=O and O–C=O bonds on the surface significantly increases, indicating an elevated degree of carbon oxidation on the GCE surface. Subsequent cathodic reduction on p-GCE leads to a reduction in C–O and C=O bonds, demonstrating the partial restoration of carbon conjugated structures in electrochemical reduction. The C–O bond content in p-GCE is 12.3%, significantly higher than that in GCE electrodes (4.9%), indicating a substantial increase in oxygen-containing functional groups on electrode surface after electrochemical polarization. This increase is attributed to the generation of active oxygen radicals during the anodic oxidation of GCE at high-potential (+1.30 V), where the electrochemical oxidation etches the sp^2^ conjugated carbon on the GCE surface, producing abundant oxygen-containing functional groups. These oxygen-containing groups can serve as electrocatalytic sites to enhance electrode performance and can also form covalent bonds with VMSF, achieving stable electrode modification with nanochannels.

Raman spectroscopy was employed to characterized GCE, GCE after anodic polarization and p-GCE. As shown in Fig. S1 (ESI†), the Raman peaks observed at wavenumbers 1349 cm^−1^ and 1592 cm^−1^ correspond to the disordered band (D-band) and the graphitic band (G-band) on the surface of GCE. This is primarily due to the in-plane vibrations of sp^2^ carbon. After anodic oxidation, it was found that the two Raman peaks were enhanced. This enhancement is due to the electrochemical oxidative etching of the sp^2^ conjugated carbon on the GCE surface, which generates oxygen-containing functional groups. This process increases the carbon defects in the lattice and the degree of carbonization of the material. Conversely, after cathodic reduction, p-GCE exhibits a corresponding decrease in the corresponding Raman peaks, attributed to the restoration of sp^2^ carbon during the reduction process. These results are consistent with the XPS characterization results.

### 3.3 Characterization of VMSF

**Fig. 3:**
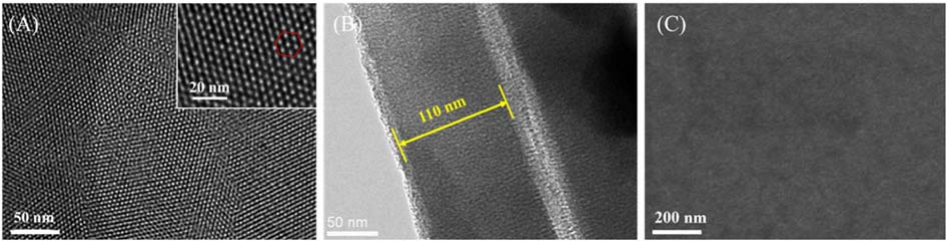
(A) Top-view TEM image of VMSF at different magnification. Inset is the corresponding high-resolution transmission electron microscope (HRTEM) image. The hexagon represents a hexagonal stacking of nanochannels. (B) TEM image of cross-section of VMSF. (C) SEM image of top-view of VMSF/p-GCE.

The morphology and structure of VMSF were characterized using transmission electron microscopy (TEM) and scanning electron microscope (SEM). Fig. 3A presents a top-view TEM image of VMSF. It can be observed that VMSF exhibits a uniformly distributed porous structure, and the film shows no defects or cracks within the observed range. The inset high-resolution TEM image reveals a hexagonally stacked mesoporous structure in VMSF. The average diameter of these nanochannels is approximately 2.8 nm, with a density of around 7.7 × 10^12^ cm^−2^, corresponding to a porosity of approximately 43%. From the TEM cross-sectional image (Fig. 3B), nanochannel could be seen and the film thickness is about 110 nm. Fig. 3C is the top-view SEM image of VMSF/p-GCE electrode. As seen, there are no obvious defects in the film.

### 3.4 Integrity and charge-selective permeability of VMSF nanochannels

**Fig. 4:**
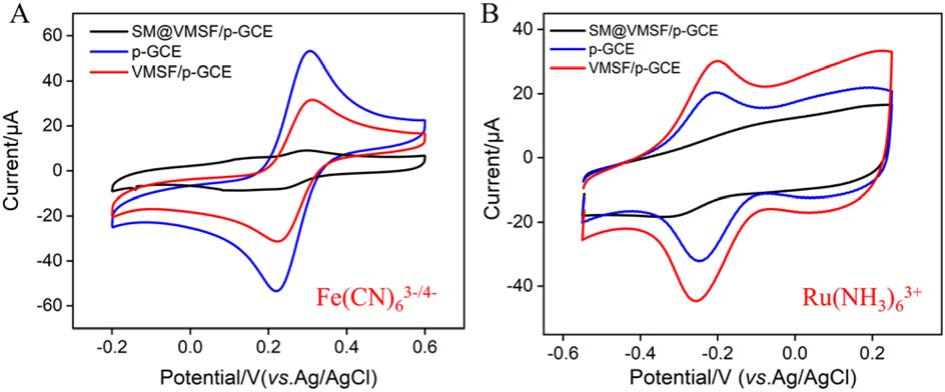
CV curves obtained on different electrodes in Fe(CN)_6_^3−/4−^ (A) or Ru(NH_3_)_6_^3+^ (B) solution.

To verify the integrity and charge-selective permeability of VMSF, oppositely charged probes, Fe(CN)_6_^3−/4−^ and Ru(NH_3_)_6_^3+^, were selected as standard redox probes, and their behavior on different electrode was investigated using cyclic voltammetry (CV). As shown in Fig. 4, when using SM@VMSF/p-GCE as the working electrode, no faradaic signals for the two electrochemical probes were detected on the electrode. This is attributed to the insulation of the underlying electrode by nanochannel film sealed with template micelles, which hinders the diffusion of electrochemical probes towards the underlying electrode. This indicates that the VMSF grown on the electrode surface has an intact and defect-free structure. Otherwise, electrochemical probes could reach the electrode surface through cracks or defects. Comparing with p-GCE, the faradaic signal of Ru(NH_3_)_6_^3+^ on VMSF/GCE, after removal of the template micelles in the nanochannels, is significantly enhanced. Conversely, the faradaic signal of the anionic probe Fe(CN)_6_^3−^/^4−^ on VMSF/GCE is noticeably suppressed, significantly lower than its current signal on the p-GCE electrode. Therefore, VMSF exhibits a remarkably charge-selective permeability to electrochemical probes of different charges. This is attributed to the silica structure of VMSF, which is rich in silanol groups with a low ionization constant (p*K*_a_ ∼ 2). The silanol group is easily ionizable in conventional media, displaying a negatively charged surface.^50–52^ The negatively charged surface induces the electrostatic adsorption towards positively charged ions and electrostatic repulsion towards negatively charged ions.^53–55^ Thus, the common negatively charged interferents such as ascorbic acid (AA) or uric acid (UA) can be significantly eliminated in the detection of Ros A.

### 3.5 Enhanced electrochemical signal of Ros A on VMSF/p-GCE

**Fig. 5:**
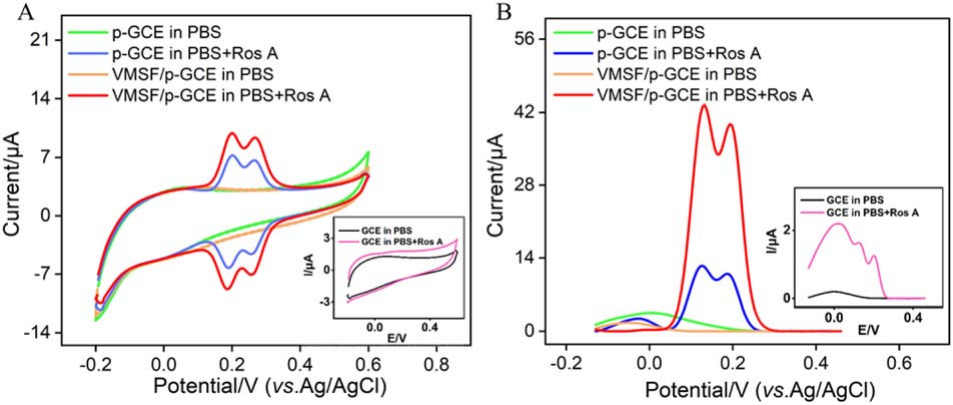
CV curves (A) and DPV curves (B) obtained on different electrodes in 0.1 M PBS (pH = 7) without or with Ros A (20 μM) at a scan rate of 50 mV s^−1^. Insets show the corresponding CV or DPV curves obtained on GCE in the presence or absence of Ros A.

The electrochemical signals of Ros A on different electrodes were investigated using CV and differential pulse voltammetry (DPV). Fig. 5A displays the CV curves obtained on different electrodes before and after the addition of Ros A. Compared to that of GCE (inset in Fig. 5A), the peak current signal of Ros A measured on p-GCE significantly increased. This is attributed to the fact that electrochemical polarization enhances the electrochemical activity of the electrode. After electrochemical activation, the p-GCE surface contains more oxygen-containing functional groups than the GCE surface, which can enhance the adsorption of Ros A through electrostatic or hydrogen bonding interactions. DPV curves display similar results (Fig. 5B). As shown in Fig. 1, Ros A is an ester of caffeic acid and 3,4-dihydroxyphenyl lactic acid, containing A and B two rings. It can undergo electrochemical oxidation in two consecutive steps. Firstly, the two hydroxyl groups in ring A undergo dehydrogenation to form a more stable free radical. Thus, the oxidation–reduction reaction occurs first on the two phenolic hydroxyl groups in ring A (0.202 V). The second oxidation step occurs at a more positive potential (0.268 V), corresponding to the oxidation of the 3,4-dihydroxyphenyl lactic acid residue. When using VMSF/p-GCE as the working electrode, the peak current of Ros A is further significantly increased. This is attributed to the signal-enhancing effect of the nanochannels. The DPV curve in Fig. 5B confirms the same conclusion. Thus, both the underlying electrode and the nanochannel modification contribute to the enhancement of the electrochemical signal of Ros A. The constructed sensor exhibits a dual signal-enhancing effect, showing potential for highly sensitive detection of Ros A. Due to the fact that the first oxidation peak occurs at a lower potential and has a higher signal, this peak was chosen for subsequent optimization and detection.^56^ An unmodified GCE was used as a control to prepare the VMSF/GCE. Fig. S2 (ESI†) displays the Ros A response on the VMSF/GCE. As seen, the peak current signal of Ros A on VMSF/p-GCE electrode increases by 166 times compared to that on VMSF/GCE. This enhancement is attributed to the electrochemical polarization which enhances the electrochemical activity of the electrode. After electrochemical activation, the p-GCE surface contains more oxygen-containing functional groups compared to the GCE, which can enhance the adsorption of Ros A through electrostatic or hydrogen bonding interactions, thereby improving the electrochemical signal. Additionally, VMSF cannot be stably bound to GCE, while VMSF on p-GCE also enhances the electrochemical response of Ros A.

### 3.6 Optimization of Ros A detection conditions

To enhance the performance in detecting Ros A, the pH conditions for p-GCE preparation, the growth time of VMSF, the electrochemical processes of Ros A on the electrode, the pH of the supporting electrolyte and the enrichment time for Ros A detection were investigated. Different solutions were used for p-GCE preparation including a strongly acidic solution (0.1 M H_2_SO_4_), acidic or neutral PBS (pH 5 or 7), and a strongly alkaline solution (0.1 M NaOH). The p-GCE prepared at each solution was then used for VMSF growth. Fig. S3 (ESI†) shows the electrochemical response of the obtained VMSF/p-GCE towards Ros A. It can be observed that the p-GCE prepared with PBS at pH 5 exhibits the highest signal for Ros A after VMSF growth. Thus, PBS at pH 5 was chosen for subsequent preparation of p-GCE. Fig. S4 (ESI†) displays DPV curves obtained in Ros A using VMSF/p-GCE electrodes fabricated on p-GCE prepared using different anodizing time. As observed, the signal for detecting Ros A using the VMSF/p-GCE prepared with an anodic oxidation time of 300 seconds is the highest. With the increase in anodic oxidation time, electrochemical oxidative etching of the sp^2^ conjugated carbon on the GCE surface occurs, generating more oxygen-containing functional groups. These groups are beneficial for enriching Ros A through electrostatic or hydrogen bond interactions, thereby enhancing the electrochemical signal. However, excessive oxidation time might lead to a decrease in the conductivity or active surface area of the electrode. Thus, an anodic oxidation time of 300 seconds was selected.

**Fig. 6:**
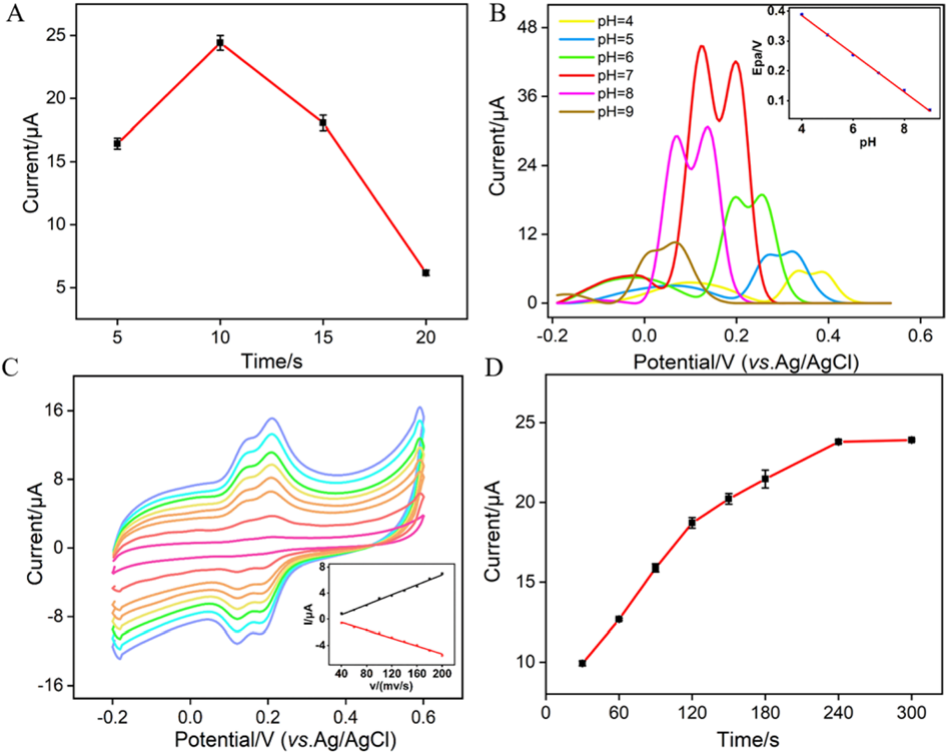
(A) The current of (10 μM) Ros A obtained on VMSF/p-GCE constructed using different VMSF growth time. (B) DPV curves obtained on VMSF/p-GCE in Ros A (20 μM) at different pH values. The inset shows the linear dependence between anodic peak potential and pH. (C) CV curves obtained on VMSF/p-GCE in PBS (0.01 M, pH = 7) containing Ros A (10 μM) at different scan rate (40, 60, 80, 100, 120, 140, 160, 180, 200 mV s^−1^ from outside to inside), the inset is the relationship between peak current and scan rate. (D) Effect of enrichment time on the current response of Ros A (10 μM) in 0.1 M PBS (pH = 7). The error bars represent the standard deviations of three measurements.

Since the thickness of VMSF is correlated with the growth time in the EASA method, Fig. 6A shows the DPV peak current for Ros A detection on electrodes with different VMSF growth time. The optimal growth time was observed to be 10 seconds. This could be attributed to the fact that, with too short of a growth time, the electrostatic adsorption effect of the nanochannels is not significant, while with an excessively long VMSF growth time, silica particles are prone to forming at the nanochannel exits, thereby affecting the diffusion of Ros A to the underlying electrode. As shown in Fig. 6B, within the pH range of 5–9, the peak potential (*E*_pa_) of Ros A exhibits a good linear relationship with the pH of the supporting electrolyte (*E*_pa_ = −0.063pH − 0.6405, *R*^2^ = 0.999). The negative shift of *E*_pa_ with increasing pH indicates the involvement of protons in the electrochemical oxidation–reduction process of Ros A. When the pH of the PBS solution is 7, the peak current signal for Ros A is the highest. In addition, the calculated proton/electron ratio (*m*/*n*) is 1.065, approximately equal to 1, indicating an equal number of protons and electrons participating in the electrochemical reaction. Fig. 6C shows the CV curves obtained at different scan rates. It can be observed that the peak potentials for oxidation and reduction remain relatively constant, indicating a high electron transfer rate of Ros A on the electrode surface. The oxidation peak current (*I*_a_) and reduction peak current (*I*_c_) both increase with increasing scan rate, showing a linear relationship with the scan rate (*I*_a_ = 0.038*v* − 0.837, *R*^2^ = 0.991; *I*_c_ = −0.030*v* + 0.725, *R*^2^ = 0.991), indicating that the electrochemical process of Ros A on the electrode is adsorption-controlled. Fig. 6D presents the oxidation peak current of Ros A detected on the electrode after different enrichment time. With increasing enrichment time, the peak current initially increases and then stabilizes at 240 s, indicating that the adsorption sites in the nanochannels reach saturation. Thus, an enrichment time of 240 s was used for subsequent experiments.

### 3.7 Electrochemical detection of Ros A

**Fig. 7:**
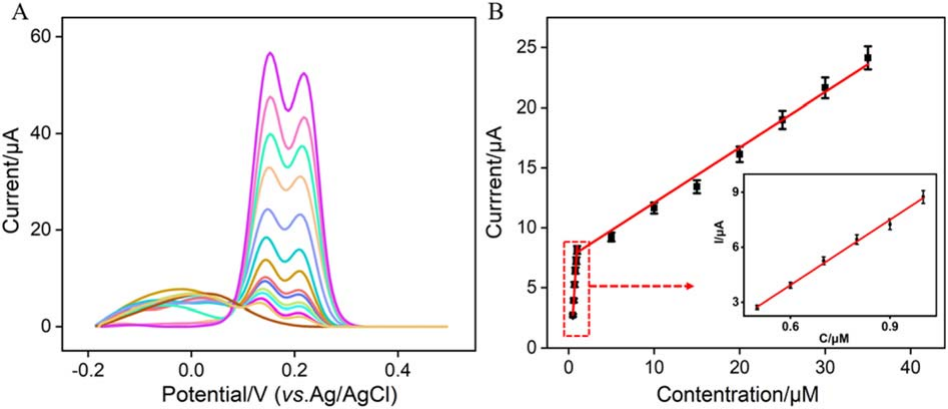
(A) DPV curves obtained on VMSF/p-GCE in 0.1 M PBS solution (pH = 7) containing different concentrations of Ros A. (B) The corresponding calibration curve between the DPV curves and the concentration of Ros A. Inset is the amplified view of the DPV curves in the low-concentration region. The error bars represent the standard deviations of three measurements.

Under the optimized conditions, electrochemical detection of Ros A was conducted using DPV. Fig. 7A displays the DPV curves obtained for different concentrations of Ros A on VMSF/p-GCE. It can be observed that as the concentration of Ros A increases, the oxidation peak current gradually increases. The peak current (*I*) exhibits a linear correlation with Ros A concentration (*C*) in the ranges from 500 nM to 1 μM (*I* = 11.4*C* − 2.91, *R*^2^ = 0.994) and 1 μM to 35 μM (*I* = 0.45*C* + 7.24, *R*^2^ = 0.990) (Fig. 7B). The detection limit (DL), calculated using a signal-to-noise ratio of 3 (S/N = 3), is determined to be 26 nM. Comparison between detection of Ros A using different method is demonstrated in Table S1 (in ESI†).^2,3,11,57–59^ DL is lower than that obtained by differential pulse voltammetry (DPV) measurement using poly(*o*-phenylenediamine)/Pt nanoparticles modified GCE (PoPD/Pt/GCE),^3^ or CV detection using graphene oxide-peptide modified screen-printed carbon electrode (GO-peptide/SPCE) or Fe_3_O_4_-phthalocyanine-carbonylated multiwalled carbon nanotubes modified magnetic glassy carbon electrode (Fe_3_O_4_-Pc-CMWCNTs/MGCE) or, Fe_3_O_4_@SiO_2_@NH_2_@magnetic molecularly imprinted polymer modified carbon paste electrodes (Fe_3_O_4_@SiO_2_@NH_2_@MMIP/CPEs),^11,58,59^ but higher than that obtained using fluorescence detection based on NaYF_4_:Yb/β-cyclodextrin modified citric acid/oxidized 3,3′,5,5′-tetramethylbenzidine (Y:Yb/Er-Cit-CD/oxTMB), or CV detection using ionic liquid/CNTs paste electrode (IL/CNTs-PE).^2,57^

### 3.8 Selectivity, anti-interference, and regeneration of the sensor

**Fig. 8:**
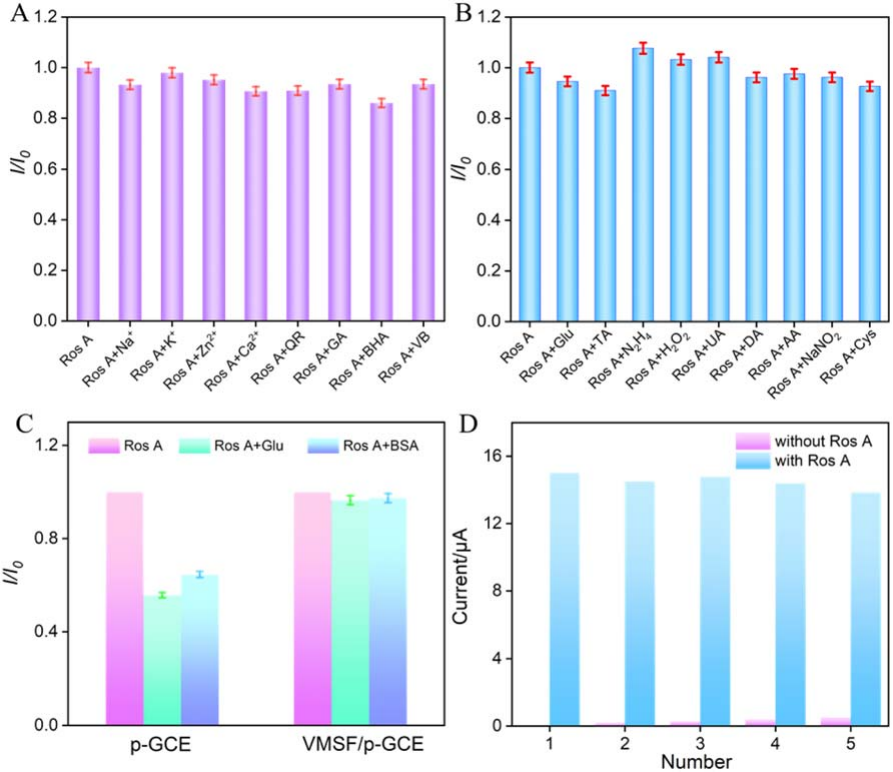
(A and B) The current ratio (*I*/*I*_0_) obtained from VMSF/p-GCE for detection of Ros A (20 μM) in the absence (*I*) and presence (*I*_0_) of the added interfering species. The concentration of interfering species is 100 μM. (C) The peak current ratio of Ros A (20 μM) on p-GCE or VMSF/p-GCE in absence (*I*_0_) or presence (*I*) of 10 μM BSA or DNA in PBS (0.1 M, pH = 7). (D) The regeneration of VMSF/p-GCE.

To assess the anti-interference capability of electrode detection for Ros A, the influence of common metal ions (Na^+^, Ca^2+^, Zn^2+^, K^+^) and polyphenolic compounds including quercetin (QR), gallic acid (GA), butylated hydroxyanisole (BHA), and vitamin B (VB) on Ros A detection was investigated. As shown in Fig. 8A, none of the above-mentioned substances, including redox-type small molecules, significantly affected the detection of Ros A, demonstrating excellent detection selectivity. The effects of common electroactive substances such as glucose (Glu), tartaric acid (TA), hydrazine (N_2_H_4_), hydrogen peroxide, uric acid (UA), dopamine (DA), ascorbic acid (AA), sodium nitrite, and cysteine (Cys) on the detection of Ros A were also investigated. As shown in Fig. 8B, none of these substances, including redox-active small molecules, significantly affected the detection of Ros A, demonstrating the excellent detection selectivity. This can be attributed to the different potentials between these substances and Ros A, as well as the electrostatic repulsion by the VMSF nanochannels towards negatively charged redox substances such as AA and UA. Additionally, the fouling resistance ability of VMSF/p-GCE was examined. As illustrated in Fig. 8C, the peak current of Ros A detected on VMSF/p-GCE remained almost unchanged in the presence of large molecular substances (protein-BSA) and DNA. In contrast, using p-GCE as the working electrode, the signal of Ros A was significantly affected by BSA or DNA, proving the excellent fouling resistance capability of the VMSF modification layer. This fouling resistance is attributed to the size exclusion effect of the ultrasmall nanochannels in VMSF.^60,61^ Moreover, the constructed sensor can be easily regenerated. The used electrode can be immersed in an HCl–ethanol solution and stirred for 10 minutes to remove residual Ros A, achieving electrode regeneration. Fig. 8D presents the peak current determined during the electrode regeneration process in different solutions. Specifically, the peak current of Ros A (20 μM) was recorded on VMSF/p-GCE, followed by electrode regeneration and measurement of the electrochemical signal on the regenerated electrode in the supporting electrolyte. This process was repeated for five cycles. It is evident that the regenerated electrode exhibits extremely low background signals in the electrolyte, and the signal for Ros A measured on the regenerated electrode is very similar to the initial signal, proving the electrode's convenient regeneration for repeated use.

### 3.9 Real sample analysis

**Fig. 9:**
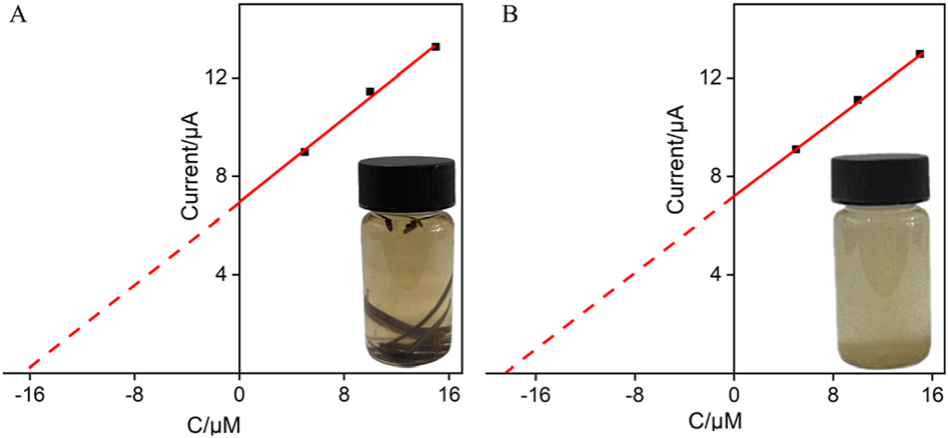
Determination of Ros A in rosemary tea (A) and leaching solution of rosemary powder (B) using the extrapolation of the standard addition method.

The good anti-fouling and anti-interference performance of VMSF/p-GCE suggests its potential in direct analysis of complex samples. The application of the constructed sensor for real sample analysis was explored using the detection of Ros A in extraction solutions of rosemary tea leaves and powder. For both rosemary tea leaves and powder extracts, 5, 10, and 15 μM of Ros A were separately added for detection. Using the extrapolation method from the standard curve (Fig. 9), the content of Ros A in rosemary tea leave was determined to be 6.7%, while it was found to be 6.6% in rosemary powder. The detected contents of Ros A were similar with that determined using the standard high-performance liquid chromatography (HPLC).

## 4 Conclusions

In summary, a method for the preparation of a simple electrochemical sensor for the detection of rosmarinic acid by integrating VMSF with highly active p-GCE was demonstrated. The p-GCE was prepared through simple electrochemical polarization of GCE. Without using any adhesives, the fast and stable integration of VMSF onto the p-GCE surface was successfully achieved using the EASA method. Both p-GCE and the nanochannel array of VMSF contributed to the electrochemical signals of Ros A, exhibiting a dual signal enhancement effect. The constructed VMSF/p-GCE demonstrated high sensitivity for electrochemical detection of rosmarinic acid. Combining the ultrasmall nanochannel array with high potential resolution of p-GCE, VMSF/p-GCE showed high selectivity and anti-interference capability. Moreover, it could be easily regenerated and reused after a simple regeneration process. This work provides a new strategy for the rapid and convenient fabrication of electrochemical sensors with high sensitivity, selectivity, and anti-fouling properties for the detection of rosmarinic acid.

## Data availability

The data presented in this study are available on request from the corresponding author.

## Author contributions

Investigation, W. Chen and H. Ru; data curation, W. Chen and H. Ru; writing – original draft preparation, W. Chen and H. Ru; writing – review and editing, F. Yan, and X. Mo; conceptualization and supervision, F. Yan, and X. Mo. All authors have read and agreed to the published version of the manuscript.

## Conflicts of interest

The authors declare that the research was conducted in the absence of any commercial or financial relationships that could be construed as a potential conflict of interest.

## Supplementary Material

RA-014-D4RA03063J-s001
