# Supplementary material for: Simple and direct electrochemical detection of rosmarinic acid in food samples based on nanochannel modified carbon electrode
Source: RSC Adv. 2024 Jun 11;14(26):18599–607. doi: 10.1039/d4ra03063j (PMC11165691; doi:10.1039/d4ra03063j)
Supplement: RA-014-D4RA03063J-s001 [file RA-014-D4RA03063J-s001.pdf]

Electronic Supplementary Material (ESI) for RSC Advances.
This journal is © The Royal Society of Chemistry 2024

Table S1. Comparison between detection of Ros A using different method.

Electrode	Method	Sample	Liner range (μM)	DL (μM)	Ref.
Y:Yb/Er-Cit-CD/oxTMB	FL	Rosemary bread	0.010-5.00	0.004	[2]
PoPD/Pt/GCE	DPV	Melissa officinallis drug plant	1.00-55.0	0.500	[3]
GO-Peptide/SPCE	CV	Cosmetic Products	0.100-3.20	0.097	[11]
IL/CNTs-PE	CV	Edible Flowers	0-680	0.015	[57]
Fe_3O_4-Pc-CMWCNTs/MGCE	CV	leach liquor of plants and Human serum	0.200- 400	0.182	[58]
Fe_3O_4@SiO_2@NH_2@ MMIP/CPEs	CV	plant extracts	0.100-500	0.085	[59]
VMSF/p-GCE	CV	rosemary tea leaves and powder	0.500-22.0	0.026	This work

Y:Yb, $NaYF_4$:Yb; Er-Cit-CD, β-cyclodextrin modified citric acid; oxTMB, oxidized 3, 3’, 5, 5’-tetramethylbenzidine; FL, fluorescence; PoPD, Poly (o-phenylenediamine); Pt, Pt nanoparticles; GCE, glassy carbon electrode; DPV, differential pulse voltammetry; GO, graphene oxide; SPCE, screen-printed carbon electrode ; CV, cyclic voltametry; IL, ionic liquid; CNTs, carbon nanotubes; CNTs-PE, CNTs paste electrode; Pc, phthalocyanine; CMWCNTs, carbonylated multiwalled carbon nanotubes; MGCE, magnetic glassy carbon electrode; MMIP, magnetic molecularly imprinted polymer; CPEs, carbon paste electrodes

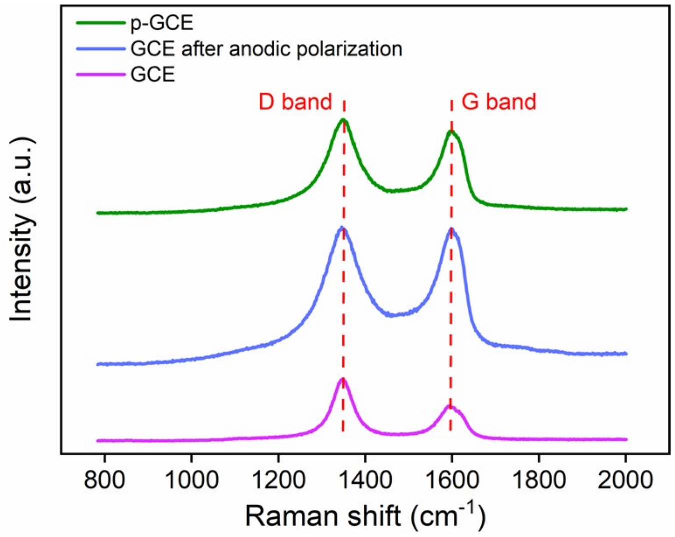

Fig. S1. Raman spectra obtained on different electrodes.

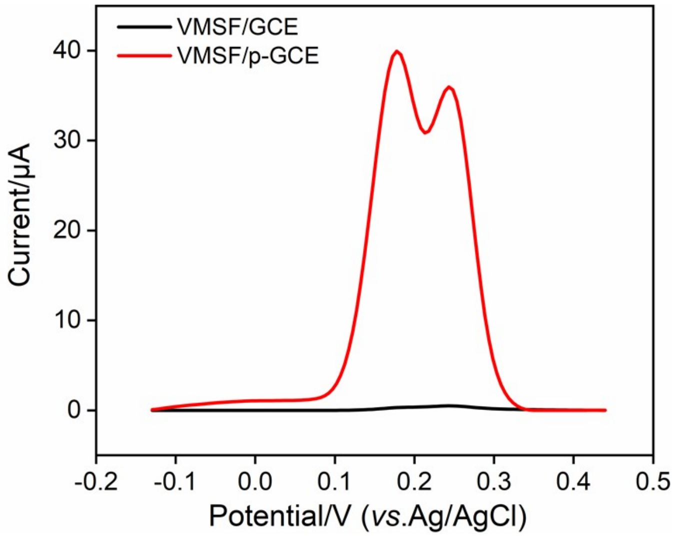

Fig. S2. DPV curves obtained on different electrodes in Ros A (20 μM) solution.

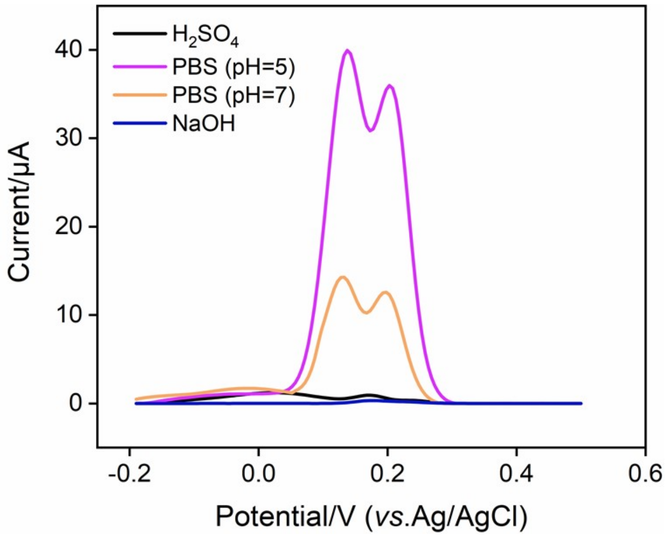

Fig. S3. DPV curves obtained in Ros A (20 μM) using VMSF/p-GCE electrodes fabricated on p-GCE prepared using different solutions.

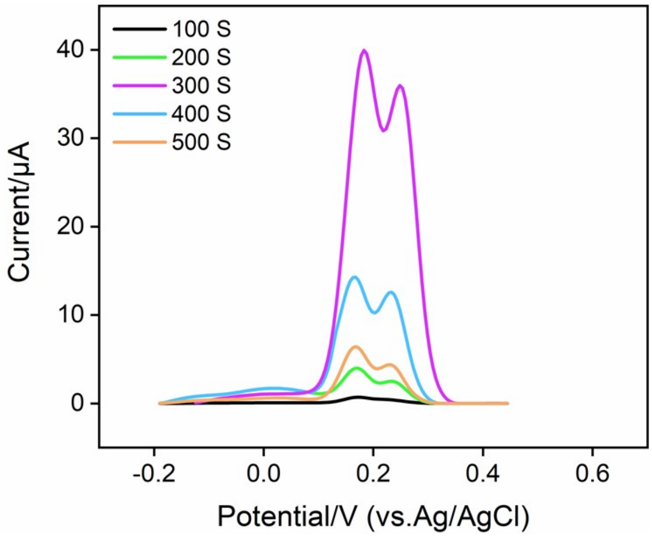

Fig. S4. DPV curves obtained in Ros A (20 μM) using VMSF/p-GCE electrodes fabricated on p-GCE prepared using different anodizing time.
